# First Case of Heterochthonous Subconjunctival Dirofilariasis Described in Poland

**DOI:** 10.4269/ajtmh.2010.10-0084

**Published:** 2010-08-05

**Authors:** Maria Wesolowska, Krystian Kisza, Marek Szalinski, Marcin Zielinski, Anna Okulewicz, Marta Misiuk-Hojlo, Beata Szostakowska

**Affiliations:** Department of Biology and Medical Parasitology, University of Medicine, Wroclaw, Poland; Department of Ophthalmology, University of Medicine, Wroclaw, Poland; Department of Parasitology, Wroclaw University, Wroclaw, Poland; Inter-Faculty Institute of Maritime and Tropical Medicine, Department of Tropical Parasitology, Medical University of Gdansk, Gdynia, Poland

A 55-year-old Polish male presented with discomfort, redness, itching, and swelling in the left eye that had begun three days before seeking medical help. Slit-lamp examination revealed an actively moving subconjunctival worm located inferotemporally (Figure 1). A complete, live, moving nematode was removed under local anesthesia, and it was first stored in physiological saline for microscopic examination and then, put into 70% alcohol with glycerin and submitted for parasitological identification. The diagnosis was based on the following morphological features of the parasite: body length = 85.1 mm; maximum body width = 0.545 mm; esophagus = 0.895 mm long; vulva a little behind the esophagus; tail = 0.10 mm long, almost terminal; and cuticle with longitudinal combs on the surface (Figure 2). The worm was identified as an immature female nematode of the species *Dirofilaria repens* (*Onchocercidae*). The patient's blood tests were within the normal limits. Neither eosinophilia nor microfilaremia were detected.

**Figure 1.**
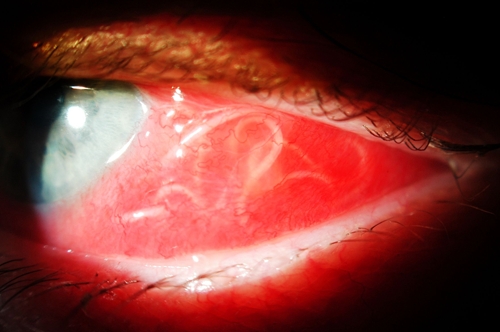
An immature female worm of *Dirofilaria repens* located subconjunctivaly with accompanying chemosis. This figure appears in color at www.ajtmh.org.

**Figure 2.**
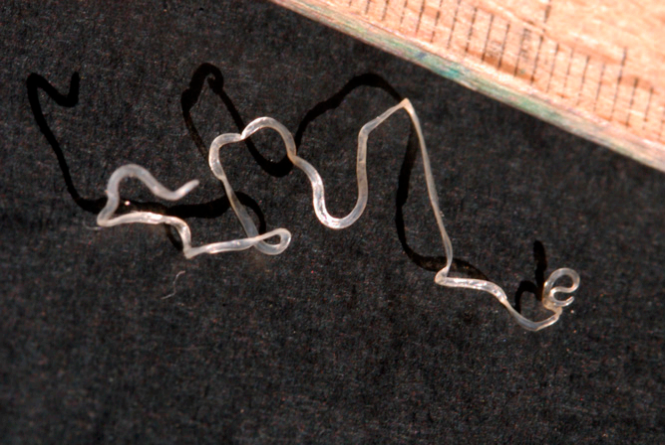
An 85.1-mm-long and 0.545-mm-wide immature female worm removed from the subconjunctival nodule. This figure appears in color at www.ajtmh.org.

The infection with ocular dirofilariasis probably occurred during a visit to Greece 8 months before the symptoms occurred.^1^ However, because of recent reports of new endemic areas of dirofilariasis in countries conterminous to Poland, an autochthonic infection cannot be excluded.^2^

The differential diagnosis of subconjunctival filariasis should include loaiasis.

## References

[1] Tzanetou K Gasteratos S Pantazopoulou A Gogou C Konidaris D Fragia K 2009 Subcutaneous dirofilariasis caused by *Dirofilaria repens* in Greece: a case report J Cutan Pathol 36 892 895 19586500 10.1111/j.1600-0560.2008.01144.x

[2] Miterpakova M Antolova D Hurníkova Z Dubinsky P 2008 Dirofilariosis in Slovakia—a new endemic area in central Europe Helmintologia 45 120 123

